# Development of the retinotectal system in the direct-developing frog *Eleutherodactylus coqui *in comparison with other anurans

**DOI:** 10.1186/1742-9994-5-9

**Published:** 2008-06-23

**Authors:** Gerhard Schlosser

**Affiliations:** 1 Brain Research Institute, University of Bremen, FB 2, P.O. Box 33 04 40, 28334 Bremen, Germany

## Abstract

**Background:**

Frogs primitively have a biphasic life history with an aquatic larva (tadpole) and a usually terrestrial adult. However, direct developing frogs of the genus *Eleutherodactylus *have lost a free living larval stage. Many larval structures never form during development of *Eleutherodactylus*, while limbs, spinal cord, and an adult-like cranial musculoskeletal system develop precociously.

**Results:**

Here, I compare growth and differentiation of the retina and tectum and development of early axon tracts in the brain between *Eleutherodactylus coqui *and the biphasically developing frogs *Discoglossus pictus*, *Physalaemus pustulosus*, and *Xenopus laevis *using morphometry, immunohistochemical detection of proliferating cell nuclear antigen (PCNA) and acetylated tubulin, biocytin tracing, and in situ hybridization for *NeuroD*. Findings of the present study indicate that retinotectal development was greatly altered during evolution of *Eleutherodactlyus *mostly due to acceleration of cell proliferation and growth in retina and tectum. However, differentiation of retina, tectum, and fiber tracts in the embryonic brain proceed along a conserved slower schedule and remain temporally coordinated with each other in *E. coqui*.

**Conclusion:**

These findings reveal a mosaic pattern of changes in the development of the central nervous system (CNS) during evolution of the direct developing genus *Eleutherodactylus*. Whereas differentiation events in directly interconnected parts of the CNS such as retina, tectum, and brain tracts remained coordinated presumably due to their interdependent development, they were dissociated from proliferation control and from differentiation events in other parts of the CNS such as the spinal cord. This suggests that mosaic evolutionary changes reflect the modular character of CNS development.

## Background

Most frogs have a characteristic biphasic life history, with a specialized aquatic larval stage and a typically terrestrial adult. Transformation of the larva into the adult takes place during metamorphosis, a period of dramatic reorganization of the body plan at the end of larval life, which involves the loss of many larval tissues, the establishment of novel adult tissues as well as complex spatial tissue rearrangements under the control of thyroid hormones [1,2]. The phylogenetic distribution of this biphasic life history indicates that it is the ancestral developmental pattern of extant anurans [3-7]. However, in several anuran lineages, the free-living larval stage has been secondarily reduced or lost resulting in direct development [8-12]. While some larval features are recapitulated within the egg in direct developers, other larval features are lost and ontogenetic trajectories are modified and abbreviated. The degree to which larval development is recapitulated or abolished differs between different lineages of direct developing frogs with frogs of the genus *Eleutherodactylus *(Leptodactylidae) showing the most radical deviations from the ancestral pattern.

**Figure 1:**
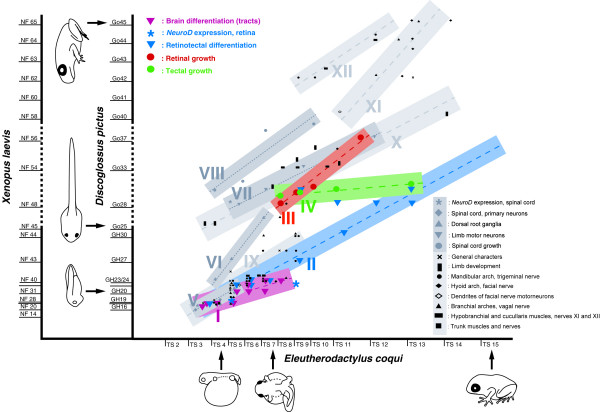
Heterochrony plot comparing the timing of retinotectal and brain development (colored symbols, suites of characters I-IV) with development of the spinal cord (grey symbols, suites of characters V-VIII) and other characters (black symbols, suites of characters IX-XII) in *E. coqui *and *D. pictus *(modified, corrected, and supplemented from [28]). X and Y axes represent time axes, along which stages of development are indicated. To facilitate comparisons, the approximate correspondence of stages of *D. pictus *[86,87] with stages of *X. laevis *[88]) are also indicated. The duration of the larval period (dashed part of Y axis) in *D. pictus *and *X. laevis *is variable and is represented here in a very telescoped fashion. All symbols in the plot except the asterisks represent developmental events (e.g., outgrowth of retinofugal fibers), whose timing in *E. coqui *is plotted against timing in *D. pictus *(for detailed list see below). The asterisks compare timing of *NeuroD *expression between *X. laevis *and *E. coqui*. Timing of events is based on data reported in this paper as well as [18,19,21,28,29]; a few data on limb development are taken from [9] and from [93] on *D. sardus*. For a detailed list of developmental events see Tables 1 and 2 (colored symbols) and references [21,28] (black and grey symbols). Whereas suites of developmental events conserved between two species compared in a heterochrony plot are expected to plot along a diagonal line, temporal dissociations (heterochronic shifts) are indicated by deviations from such a pattern [21]. The distribution of events in this heterochrony plot reveals multiple heterochronic shifts of developmental events between *E. coqui *and *D. pictus*. While retinotectal differentiation (blue triangles; suite II) remains temporally coordinated with early development of the CNS (pink triangles; suite I), early differentiation of the spinal cord (grey squares; suite V), and early embryonic development of many cranial structures (black symbols; suite IX) in *E. coqui*, the formation of lateral motor columns and innervation of the limbs (grey triangles; suite VII) is predisplaced into early embryonic stages paralleling precocious onset of limb development (black symbols; suite X) in *E. coqui*. The formation of dorsal root ganglia also occurs earlier in *E. coqui *(grey triangles; suite VI). Growth of the retina (red circles; suite III), the tectum (green circles; suite IV), and the spinal cord (grey circles; suite VIII) all occur relatively early in *E. coqui*. Metamorphic remodeling of cranial structures (black symbols; suites XI and XII) occurs immediately after embryonic cranial development (black symbols; suite IX) in *E. coqui*.

In *Eleutherodactylus*, including the particularly well studied Puerto Rican species *E*. *coqui*, many larval structures of the epidermis, the nervous system, the musculoskeletal system, and the inner organs never develop, while an adult-like cranial skeleton together with its associated muscles and nerves develops precociously [9,11,13-21]. Limb buds, which develop in larval stages of biphasically developing frogs, form already in early embryonic stages of *E. coqui *and this is paralleled by accelerated growth of the spinal cord and precocious development of dorsal root ganglia and lateral motor columns in the spinal cord, which provide the sensory and motor innervation of limbs, respectively [18,19,22-28]. Interestingly, another region of the central nervous system (CNS), the neural retina, also displays accelerated embryonic growth in *E. coqui *even though this is not accompanied by precocious differentiation of retinal cell layers [29]. As a consequence of these changes in timing (heterochronies) of various developmental events, embryos of *E. coqui *at any given stage present a complex mosaic of traits, some corresponding to embryonic stages of development of biphasically developing frogs, others corresponding to larval, metamorphic or adult stages (Fig. 1, Tables 1, 2).

**Table 1:** Schedule of retinotectal development in different anurans

	*E. coqui*	*D. pictus*	*P. pustulosus*^1^	*X. laevis*^2^
***NeuroD *expression **(Fig. 1: blue asterisks)				
Onset of *NeuroD *expression in retina	TS 5	?	?	NF 23
*NeuroD *expression downregulated in central retina	TS 9	?	?	NF 35–36
**Retinotectal differentiation **(Fig. 1: blue triangles)				
Optic vesicle	TS 3+	GH 18	≤GH24	NF 21–22^6^
Tectofugal fibers	TS 5	GH 22	?	?
Optic vesicle forms cup	TS 5	GH 22	≤GH24	NF 26–27^6^
Lens forms	TS 5	GH 22	≤GH24	NF 32^6^
Retinofugal fibers grow out	TS 6	GH 22	GH 22	NF 29–30^6^
First cells differentiate in retina (PCNA -)	TS 6	GH 23	≤GH24	NF 28–29^6,7^
Retinofugal fibers in optic chiasm	TS 7	GH 23	GH 23	NF 32^6^
Retinofugal fibers reach contralateral tectum	TS 7	GH 23	?	NF 35–37^6^
Inner plexiform layer in retina	TS 9	GH 27	GH 27	NF 35–37^6^
First cells differentiate in tectum (PCNA-)	TS 9	GH 27	GH 24	NF 41–45^9,10^
Outer plexiform layer in retina	TS 11	GH 30	Go 27	NF 36–38^6^
Tectal layers 7–9	TS 11	Go 28	Go 27	NF 48^11^
Tectal layer 5	TS 13	Go 28	Go 27	NF 48^11^
Retinal layer of outer segments of photoreceptors	TS 12	Go 28	Go 27	NF 43^8^
First ipsilateral retinofugal fibers	TS 9	Go 30	?	NF 54–55^12^
Neuropils in contralateral thalamus	TS 13	Go 30	?	NF 35–36^13^
Tectal layers 1–4^3^	TS 15+6	Go 42	Go 41	NF 51^9^
Retinofugal fibers cover contralat. tectum completely^3^	TS 15+12	Go 45	?	NF 66^8^
**Retinal growth**^4 ^(Fig. 1: red circles)				
40 %	TS 8	Go 28	Go 28–41	?
60 %	TS 9	Go 29–30	Go 28–41	?
75 %	TS 10	Go 30–31	Go 28–41	?
90 %	TS 11+	Go 37	Go 28–41	?
**Tectal growth**^4 ^(Fig. 1: green circles)				
40 %	TS 8	Go 29/	Go 28–41	?
60 %	TS 9	Go 29–30	Go 28–41	?
75 %	TS11	Go 30–31	Go 28–41	?
90 %	TS 13-	Go 31	Go 28–41	?

^1 ^First stages analyzed: GH 20 (wholemounts), GH 24 (sections)

^2 ^Except for *NeuroD *expression, data are taken from the literature

^3 ^Out of range of Figure 1

^4 ^Size of cellular layers in percent of reference size; reference size was size of cellular layers at onset of metamorphic climax (stage Go 40) in *D. pictus *and at hatching (stage TS 15) in *E. coqui*. For *P. pustulosus *only stages Go 28 and Go 41 were analyzed and size of cellular layers at stage Go 41 was used as reference

^5 ^TS 15+6 and TS 15+12 refer to stages at 6 and 12 days posthatching in *E. coqui*

^6 ^[62]

^7 ^[59]

^8 ^[58]

^9 ^[55]

^10 ^[68]

^11 ^Schlosser (unpublished observation)

^12 ^[66]

^13 ^[94]

**Table 2:** Schedule of development of brain tracts and commissures in different anurans^1 ^. (Fig. 1: pink triangles)

	*E. coqui*	*D. pictus*	*P. pustulosus*^2^	*X. laevis*^3^
Neural tube closure	TS 3	GH 16–17	≤GH 20	NF 20^4^
Rhombomeres	TS 3+	GH 18	?	?
Tract of postoptic commissure (TPOC)	TS 4	GH 18	≤GH 20	NF 24^5,6^
Postoptic commissure (POC)	TS 5	GH 20	≤GH 20	NF 28^5^
Ventral longitudinal tract (VLT)	TS 5	GH 18	≤GH 20	NF 26^6^
Descending tract of V (TV)	TS 5	GH 18	≤GH 20	NF 26^6^
Descending tract of VIII (TVIII)	TS 5	GH 20	≤GH 20	?
Tract of anterior commissure (TAC)	TS 6	GH 20	GH 22	NF 30^6^
Anterior commissure (AC)	TS 7	GH 20	GH 22	NF 32^6,7^
Supraoptic tract (SOT)	TS 6	GH 20	GH 20	NF 32^6,7^
Tract of posterior commissure (TPC)	TS 6	GH 22	GH 22	NF 32^6,7^
Posterior commissure (PC)	TS 6	GH 22	GH 24	NF 32^6,7^
Tract of commissure of posterior tuberculum (TCPT)	TS 6	GH 22	GH 22	?
Commissure of posterior tuberculum (TCPT)	TS 6	GH 22	GH 22	?
Tract of ventral tegmental commissure (TVTC)	TS 6	GH 22	GH 22	NF 32^7^
Ventral tegmental commissure (VTC)	TS 6	GH 22	GH 22	NF 32^7^
Tract of cerebellar commissure (TCC)	TS 6	GH 20	GH 22	?
Cerebellar commissure (CC)	TS 7	GH 23	GH 24	?
Dorsoventral diencephalic tract (DVDT)	TS 6	GH 20	GH 22	NF 28^5,6^
Tract of habenular commissure (THC)	TS 8	GH 23	GH 24	NF 33/34^6^
Habenular commissure (HC)	TS 8	GH 23	GH 24	?
Intertectal commissure (ITC)	TS 9^8^	GH22^8^	GH 27–29	?

^1 ^Terminology for embryonic fiber tracts based on [38,39]

^2 ^First stage analyzed: GH 20

^3 ^Data taken from literature

^4 ^[88]

^5 ^[40]

^6 ^[42]

^7 ^[41]

^8 ^Only single fibers crossing midline at these stages; not included in Figure 1.

Despite these ontogenetic modifications, *E. coqui *retains some sort of cryptic metamorphosis [11,30]. During the last third of development prior to hatching (stages TS 12 – TS 15), its musculoskeletal system and several other tissues are remodeled in a way resembling metamorphic reorganization in biphasic developers. Moreover, these events are thyroid hormone dependent: they occur immediately after the thyroid axis becomes functional in *E. coqui *(as indicated by maturation of the thyroid gland and upregulation of thyroid hormone receptors TRβ) and can be arrested when thyroid hormone synthesis is blocked [11,30,31].

In both limb and cranial muscle development, precocious development of target structures in *E. coqui *is accompanied by a correspondingly precocious development of their innervation. The present paper addresses the question, whether the differentiation of the retina in *E. coqui *also remains closely temporally coordinated with the differentiation of the major central target of retinofugally projecting fibers in frogs, the optic tectum, and how this relates to the differentiation of other parts of the brain. Furthermore, it explores, whether growth of the optic tectum is accelerated like growth of the retina in *E. coqui *compared to the ancestral pattern of biphasically developing frogs.

In order to address these questions, development of retina and tectum is compared between *E. coqui *and the biphasically developing frogs *Discoglossus pictus *(Discoglossidae) and *Physalaemus pustulosus *(Leptodactylidae). While retinotectal projections have been studied in adult *D. pictus *[32], its embryonic development has not been previously reported. *D. pictus *was chosen as a representative of a basal anuran family with biphasic development. *P. pustulosus *was chosen, because it is relatively closely related to *E. coqui *(both frogs belong to the Leptodactylidae) but retains biphasic development. Comparison between the biphasic pattern of development in *D. pictus*, *P. pustulosus*, and other previously studied biphasically developing anurans (e.g. *Xenopus*) allows to identify shared features, which likely reflect the ancestral pattern of anuran development, from which direct development in *Eleutherodactylus *was derived. In addition, the expression of the neuronal differentiation gene *NeuroD *[33,34] in retina and tectum was compared between *E. coqui *and *Xenopus laevis *(Pipidae), since this gene has not been cloned from *D. pictus *or *P. pustulosus*.

The present study shows that in *E. coqui *embryonic growth of the optic tectum is accelerated paralleling rapid growth of the retina, while differentiation of tectal layers and development of the retinotectal projection proceeds according to a much slower schedule in coordination with retinal differentiation and the differentiation of fiber tracts in the brain. Thus, *E. coqui *hatchlings resemble postmetamorphic froglets of biphasically developing frog species with respect to differentiation of cranial muscles, limbs and spinal cord, and the size of their brains, but resemble tadpoles with respect to differentiation of their retinotectal system. These findings suggest that the schedule of differentiation of directly interconnected parts of the CNS such as retina and tectum remains coordinated during evolution, but can evolve largely decoupled from growth control and from the differentiation of other parts of the CNS (e.g. spinal cord).

## Results

In the following, I will frequently refer to *E. coqui *hatchlings (at stage TS 15) and compare them with various developmental stages in biphasically developing frogs. As a note of clarification, I should point out that this is done without intending to attach any particular significance to the time of hatching itself. The latter is highly variable between species and has little developmental significance. *E. coqui *hatchlings are rather singled out for comparison because with respect to many characters (e.g., musculoskeletal system, tail regression, limbs, brain size) they have reached a developmental stage, which corresponds to biphasically frogs at the completion of metamorphosis.

### Differentiation and growth of retina and optic tectum

**Figure 2:**
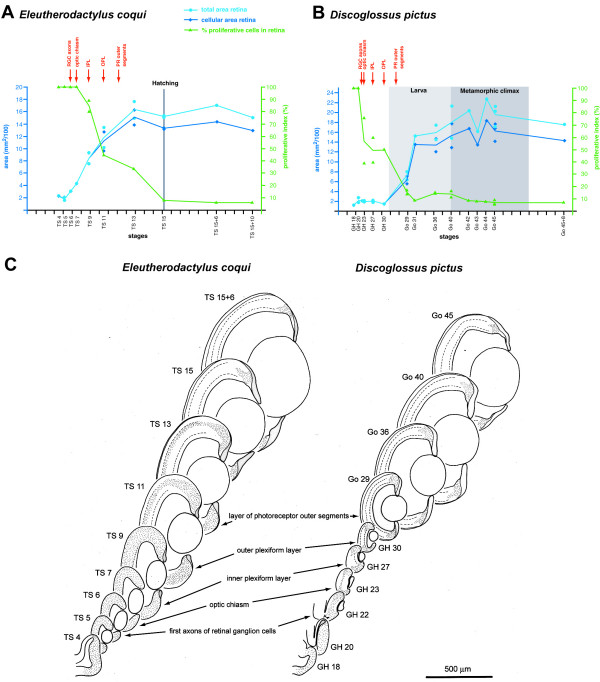
**Retinal development in *E. coqui *and *D. pictus***. (**A**, **B**) Morphometric analysis of retinal development (modified from [29]). Cross-sectional area of the central retina (light blue) or of its cellular layer (dark blue) is plotted against developmental age. In addition, a proliferative index indicating the PCNA positive proportion of the cellular layer is shown in green. Each symbol represents a measurement from a single individual (there are less data points for the proliferative index than for area measurements because PCNA staining was not apparent in each individual). Curves are drawn through mean values in case more than one individual per stage was analyzed. For each species, timing of first outgrowth of axons from retinal ganglion cells (RGC), the first retinofugal fibers in the optic chiasm, the formation of inner and outer plexiform layers (IPL and OPL, respectively) and of a distinct layer of photoreceptor (PR) outer segments in the retina are indicated in red. For *D. pictus *the approximate duration of larval and metamorphic phases are emphasized, while for *E. coqui*, which lacks a free-living larva, the time of hatching is shown (at hatching, *E. coqui *corresponds to frogs at the end of metamorphosis with respect to many characters). In each graph, one scale unit of the abscissa represents 1 day of development at 24°C, except for larval stages of *D. pictus*, which are of variable duration and are represented here in an extremely telescoped way. TS 15+6, TS 15+10 and Go 45+8 refer to stages at 6 and 10 days posthatching (*E. coqui*) or 8 days postmetamorphosis (*D. pictus*), respectively. (**C**) Overview of retinal development in *E. coqui *and *D. pictus *based on camera lucida drawings of sections through the central retina of different developmental stages. Hatched lines indicate plexiform layers, the inner nuclear layer is sandwiched in between. Stippling indicates PCNA-positive regions.

**Figure 3:**
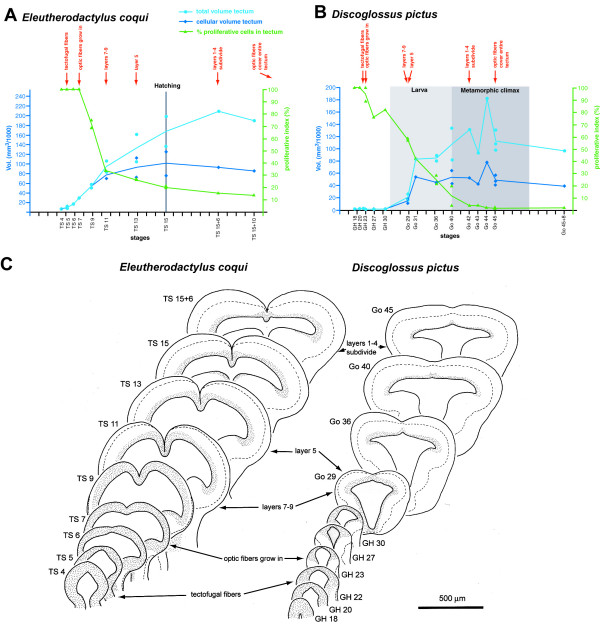
**Tectal development in *E. coqui *and *D. pictus*.** (**A**, **B**) Morphometric analysis of tectal development. Volume of the entire optic tectum (light blue) or of its cellular layer (dark blue) is plotted against developmental age. In addition, a proliferative index indicating the PCNA positive proportion of the cellular layer is shown in green. Each symbol represents a measurement from a single individual (there are less data points for the proliferative index than for area measurements because PCNA staining was not apparent in each individual). Curves are drawn through mean values in case more than one individual per stage was analyzed. For each species, timing of first outgrowth of tectofugal fibers, first ingrowth of optic (retinofugal) fibers into the tectum, the formation of tectal layers 7–9, 5, and 1–4, and the coverage of the entire surface of the tectum by optic fibers is indicated in red. Phases of development are depicted as described in Fig. 2. (**C**) Overview of tectal development in *E. coqui *and *D. pictus *based on camera lucida drawings of sections through the central tectum of different developmental stages. Hatched line indicates border between cellular layers adjacent to the ventricle and peripheral fiber layers. Within the tectum, the hatched line indicates border between layers 1–6 (mostly cellular) and layers 7–9 (mostly fibers). Stippling indicates PCNA-positive regions.

Development of retina and tectum were analyzed and their size was measured in paraffine sections of various developmental stages of *E. coqui*, *D. pictus*, and *P. pustulosus *ranging from neural tube closure to postmetamorphic (posthatching in *E. coqui*) stages. Because the size of retina and tectum reaches a plateau at the beginning of metamorphic climax in *D. pictus *(stage Go 40) and *P. pustulosus *(stage Go 41) and around the time of hatching (stage TS 15) in *E. coqui *(Figs. 2, 3), these plateau values are taken as reference size for each species. Retinal and tectal size at each stage are expressed as fraction of reference size (size/reference size), which facilitates comparison of retinal and tectal growth between species (Fig. 1, Table 1).

The development of the retina in *E. coqui *and *D. pictus *has been previously described [29]. Briefly, in *E. coqui*, outgrowth of the first retinofugal fibers and differentiation of the various cell and fiber layers of the retina during embryonic development is accompanied by rapid retinal growth (Fig. 2A, C). In contrast, in *D. pictus*, retinofugal fibers grow out and retinal layers form during embryonic development, preceding a period of prolonged growth of the retina during subsequent larval development (Fig. 2B, C). Similar to *D. pictus*, the formation of different retinal layers in *P. pustulosus *(data not shown) is also completed during embryonic development (with formation of a layer of photoreceptor outer segments at Go 27) prior to retinal growth during larval stages. At the stage when retinal layer formation is completed, the retinae of *D. pictus *and *P. pustulosus *have reached only about one third of their size at metamorphic climax, whereas the retina of *E. coqui *has reached more than 90 % of its size at hatching.

The development of the optic tectum shows similar differences between species. In the mature anuran tectum, 9 layers can be distinguished, numbered from 1 on the ventricle to 9 on the surface [35]. During early development of *E. coqui *the optic tectum consists of a single cellular layer, which increases rapidly in size (Fig. 3A, C, 4A–C). A superficial fiber layer, which will subsequently develop into layers 7–9 (two fiber layers with an interspersed layer of scattered cell bodies), is first evident at stage TS 11. At stage TS 13, when the tectum has reached more than 90 % of its size at hatching, a second fiber layer (layer 5) appears within the cellular layer, dividing the latter in a peripheral (layer 6) and ventricular part. Subdivision of this ventricular layer into distinct layers 1–4 (an ependymal layer and two cellular layers with a fiber layer sandwiched in between) becomes, however, only evident six days after hatching.

**Figure 4:**
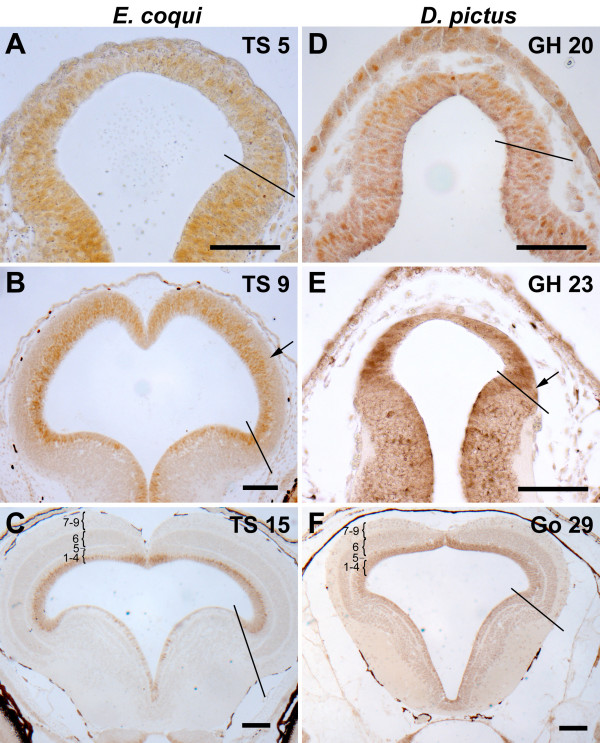
**Cell proliferation during tectal development in *E. coqui *(**A**-**C**) and *D. pictus *(**D**-**F**) as revealed by immunostaining for proliferating cell nuclear antigen (PCNA) in transverse paraffine sections**. The border of the optic tectum is indicated on the right side of each panel. Tectal layers are identified by numbers in panels **C **and **F**. At early embryonic stages of both species (**A**, **D**), the optic tectum is very small and all tectal cells are PCNA immunoreactive (orange or brown nuclei). At later embryonic stages (**B**, **E**), the tectum of *E. coqui *has enormously grown in size, while the tectum of *D. pictus *is still small (note different magnification). While the entire ventricular layer remains PCNA immunoreactive, the first non-proliferating cells are evident (arrows). At hatching, the tectum of *E. coqui *(**C**) resembles the tectum of *D. pictus *at an early larval stage (**F**): tectal layers 5 and 7–9 have differentiated, while PCNA immunopositive cells continue to be present along the entire ventricular surface. Bar: 100 μm in all panels.

In contrast, the optic tectum remains small throughout embryonic development of *D. pictus *(Fig. 3B, C, 4D–F)) and *P. pustulosus *(data not shown). The superficial (layers 7–9) and deep (layer 5) fiber layers appear at early larval stages (Go 29 in *D. pictus*, Go 27 in *P. pustulosus*), when the tectum has reached only a small fraction (about one third in *D. pictus*, less than one fourth in *P. pustulosus*) of its size at the end of metamorphic climax. Subdivision of the ventricular cell layer into layers 1–4 becomes first apparent during metamorphic climax (Go 41–42) in both species. Thus, at hatching the tectum of *E. coqui *(Fig. 4C) resembles the tectum of *D. pictus *at early to mid larval stages (Fig. 4F) with respect to the differentiation of tectal layers.

### Cell proliferation in retina and optic tectum

Proliferating cells were visualized in paraffine sections of *E. coqui*, *D. pictus *and *P. pustulosus *by immunostaining for proliferating cell nuclear antigen (PCNA), which is transiently expressed in the S phase of the cell cycle [36]. Representative stages are depicted in Figure 2, 3, 4, while the proliferative index, which measures the proportion of PCNA positive cells in the cellular layer, is plotted in Figures 2 and 3.

The time course of PCNA immunostaining in the retina of *E. coqui *and *D. pictus *has been previously described [29]. Briefly, in *E. coqui *PCNA immunopositive cells occur throughout the outer part of the inner nuclear layer of the retina until the latter has almost reached its size at hatching and only then become restricted to the ciliary margin (Fig. 2A, C). In contrast, in *D. pictus *(Fig. 2B, C) and *P. pustulosus *(data not shown), PCNA positive cells become restricted to the ciliary margin already at midembryonic stages (GH 23–27 in both species), preceding the period of larval retinal growth.

In the optic tectum of early embryos of *E. coqui *(Figs. 3A, C, 4A), *D. pictus *(Figs. 3B, C, 4D), and *P. pustulosus *(data not shown), PCNA immunopositive cells occupy the entire cellular layer. The first PCNA immunonegative and presumably postmitotic cells are seen in the lateral part of the cellular layer at stages TS 9 in *E. coqui *(Fig. 4B) and at stages GH 23–27 in *D. pictus *(Fig. 4E) and *P. pustulosus*. PCNA immunostaining is maintained along the entire ventricle (Fig. 4C, F) at least until 6 days after hatching in *E. coqui *and until the end of metamorphic climax (Go 45) in *D. pictus *and *P. pustulosus*.

### *NeuroD *expression in retina and optic tectum

**Figure 5:**
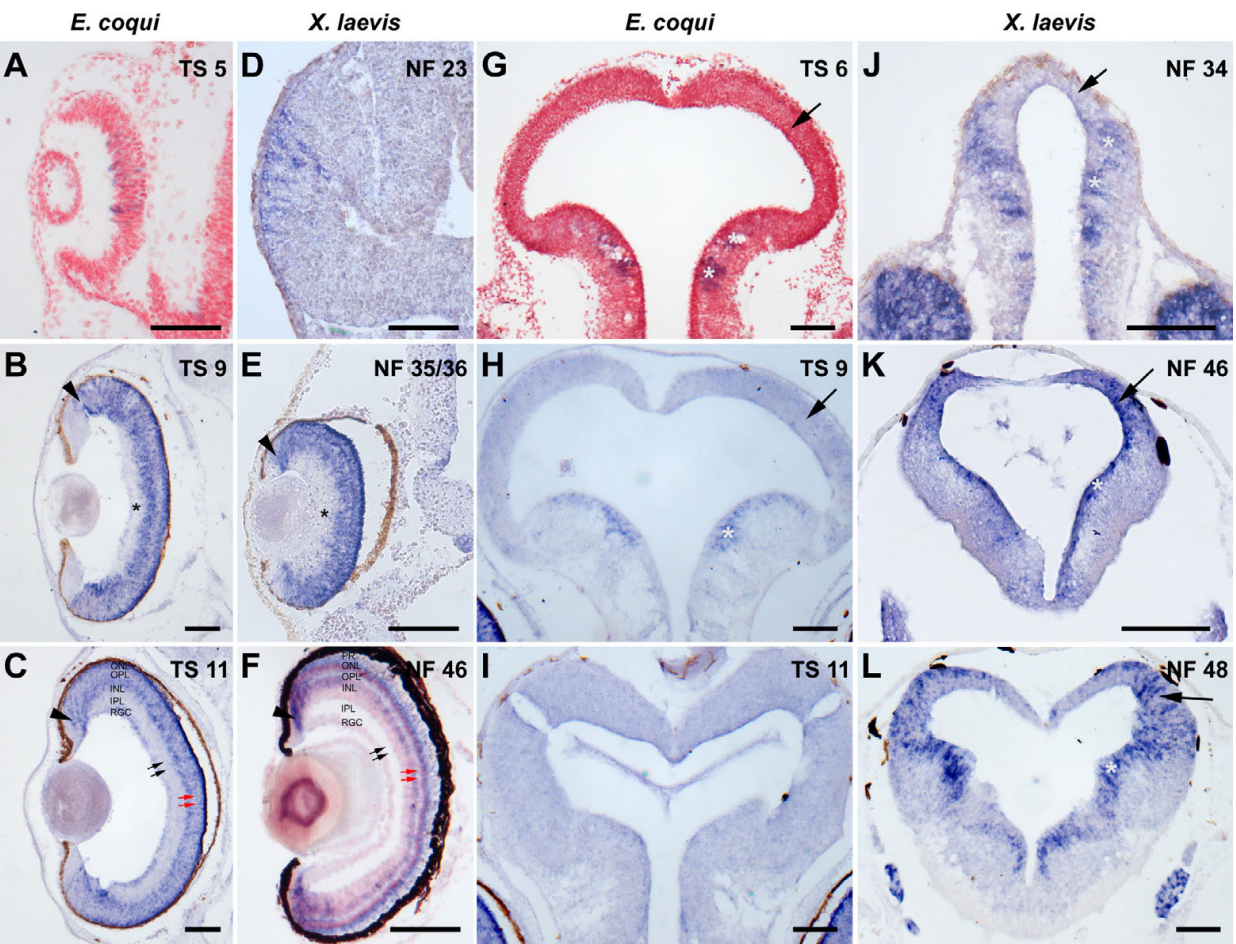
**Neurogenesis in the retina and tectum of *E. coqui *(**A**-**C, G-I**) and *X. laevis *(**D-F, J-L**) as revealed by in situ hybridization for *NeuroD *in transverse paraffine sections**. **A-F**: At early embryonic stages, *NeuroD *begins to be expressed in scattered cells throughout the retina in both species (**A**, **D**). Subsequently, most retinal cells express *NeuroD*, until *NeuroD *is downregulated in the central, inner part of the retina (asterisks) at midembryonic stages of both species (**B**, **E**). The retina of *E. coqui *has grown to much larger size at this stage than the retina of *X. laevis *(note different magnification). At later stages, *NeuroD *expression is restricted to the ciliary margin (arrowheads), the outer part of the inner nuclear layer and the outer nuclear layer in both species (**C**,**F**) and to scattered cells in the retinal ganglion cell layer in *E. coqui*. The inner and outer plexiform layers (black and red arrows, respectively) are much thinner in *E. coqui *than in *X. laevis*. **G-L**: In contrast to *X. laevis *(**J**-**L**), in which the tectum (arrows) expresses *NeuroD *from stage NF 46 on, the tectum of *E. coqui *never shows strong *NeuroD *expression at any stage (**G**-**I**) although expression is evident in other parts of the midbrain similar to *X. laevis *(asterisks). Abbreviations: INL: inner nuclear layer; IPL: inner plexiform layer; ONL: outer nuclear layer; OPL: outer plexiform layer; PR: layer of photoreceptor outer segments; RGC: retinal ganglion cell layer. Bar: 100 μm in all panels.

In order to compare the time course of neuronal differentiation in retina and tectum between *E. coqui *and a frog species with biphasic development, expression of the neuronal differentiation gene *NeuroD *was analyzed in *E. coqui *and *X. laevis *by in situ hybridization on paraffine sections of various developmental stages (Fig. 5). In *E. coqui*, *NeuroD *begins to be expressed in scattered retinal cells at stage TS 5, when the retina has already formed an optic cup and the lens vesicle has detached (Fig. 5A). At subsequent stages, *NeuroD *expression extends throughout the retina. From stage TS 9 on, when the inner plexiform layer becomes apparent, *NeuroD *continues to be expressed at the ciliary margin, the outer part of the inner nuclear layer, the outer nuclear layer and in scattered cells of the retinal ganglion cell layer, but is downregulated in the inner central part of the retina (Fig. 5B, C). In *X. laevis*, onset of *NeuroD *expression in scattered cells is already observed at stage NF 23, when the retina has not yet invaginated and no lens vesicle has formed (Fig. 5D). Subsequently, *NeuroD *expression extends throughout the retina until the gene is downregulated in the inner central parts of the retina at stage NF 35/36 (Fig. 5E) simultaneous with the appearance of the inner plexiform layer. In larval stages, *NeuroD *expression is maintained at the ciliary margin, the outer part of the inner nuclear layer and the outer nuclear layer, but not in the retinal ganglion cell layer (Fig. 5F) confirming a previous study [37].

In the optic tectum of *E. coqui*, *NeuroD *was never strongly expressed at any stage of development, although expression was evident in other regions of the midbrain and elsewhere in the brain (Fig. 5G–I). In contrast, while the embryonic tectum of *X. laevis *also does not express *NeuroD*, tectal *NeuroD *expression is upregulated from early tadpole stages (NF 46) on (Fig. 5J–L).

### Development of optic projections

**Figure 6:**
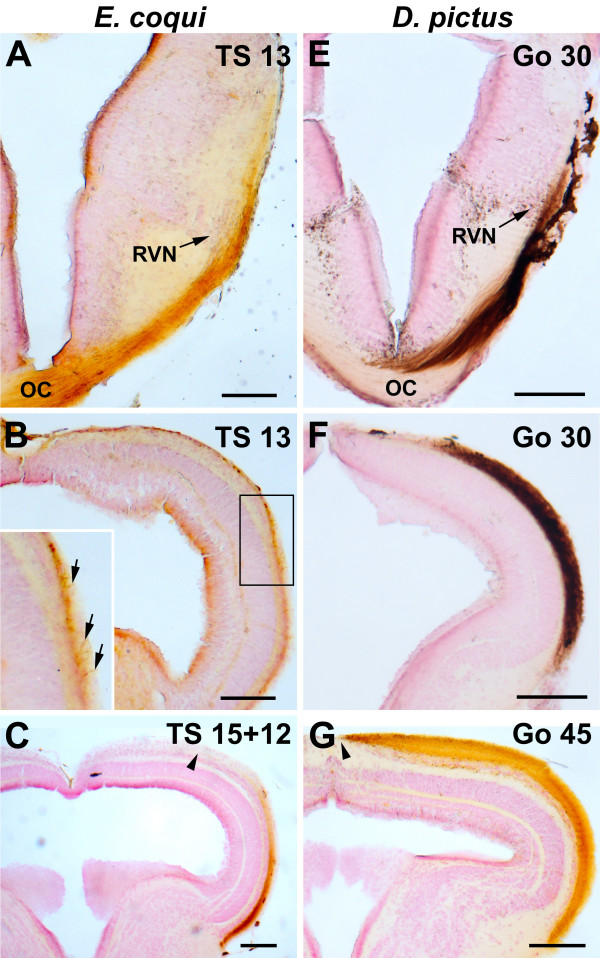
**Contralateral retinotectal projections in *E. coqui *(**A**-**C**) and *D. pictus *(**D-F**) as revealed by biocytin tracing**. At two days before hatching, the optic projections of *E. coqui *(**A**, **B**) resemble early larvae of *D. pictus *(**E**, **F**). After crossing in the optic chiasm (OC) the majority of retinofugal fibers forms the marginal optic tract, which courses dorsally in the thalamus and forms a first terminal field in the rostral visual nucleus (RVN; this probably corresponds to the nucleus lateralis of [32]) (**A**, **E**). Retinofugal fibers extend further until the optic tectum and cover approximately its rostral half (**B**, **F**). In *E. coqui*, the density of optic fibers on the tectal surface (see arrows in magnified inset of **B**) is much sparser than in *D. pictus*. At 12 days after hatching, optic fibers have reached the caudal end of the tectum in *E. coqui*, but do not yet extend towards its midline (**C**: section through midtectum; arrowheads indicate medial limit of coverage). In contrast, optic fibers cover the entire tectum in *D. pictus *at the end of metamorphic climax (**G**: section through midtectum). Bar: 100 μm in all panels.

The development of the retinofugal projection in *E. coqui *and *D. pictus *(Fig. 6, Table 1) was analyzed in serial sections after unilateral application of biocytin to the optic nerve at various developmental stages as well as in wholemount brains immunostained for acetylated tubulin to visualize neurites. The optic projection of both species conforms to the pattern observed in other frogs and develops in a similar fashion. Therefore, a detailed anatomical description of its development will not be provided and only the timing of various events in both species will be noted.

In *E. coqui*, the first neurites of retinal ganglion cells grow out at stage TS 6. These fibers cross to the contralateral side of the diencephalon in the optic chiasm at stage TS 7. The majority of labeled fibers there form the marginal optic tract, which courses dorsad. The first fibers reach the contralateral optic tectum already at stage TS 7. By stage TS 11 retinofugal fibers are sparsely distributed over the lateral part of approximately the rostral half of the tectal surface (layer 9) (Fig. 6A, B), while the caudal tip is reached only 12 days after hatching. However, even 12 days after hatching the medial part of the tectum is still free of retinofugal fibers (Fig. 6C). Terminal fields in the contralateral thalamus (rostral visual nucleus, neuropil of Bellonci, corpus geniculatum thalamicum) and pretectum (uncinate field) develop from stage TS 13 on. A much smaller number of fibers courses caudad in the contralateral basal optic tract from stage TS 11 on and terminates in the basal optic neuropil in the tegmentum. In addition to the contralateral projections, a few retinofugal fibers are evident in the ipsilateral marginal optic tract from stage TS 9 on, but no prominent ipsilateral retinothalamic projections have developed even at 12 days after hatching.

In *D. pictus*, outgrowth of the first neurites of retinal ganglion cells occurs at stage GH 22 and these fibers cross in the optic chiasm, reach the contralateral marginal optic tract and extend towards the optic tectum by stage GH 23. At early larval stages (Go 30), the surface of the lateral part of the rostral half of the tectum is densely covered by retinofugal fibers (Fig. 6E, F). Retinofugal fibers reach the caudal limit of the tectum at stage Go 40 and extend towards the midline along the entire rostrocaudal extent of the tectum at the end of metamorphic climax (Go 45) (Fig. 6G). Terminal fields in the contralateral thalamus and pretectum and a small basal optic tract are evident from early larval stages (stage Go 30, possibly already GH 30) on. A few fibers in the ipsilateral marginal optic tract are also observed from stage Go 30 on.

Applications of biocytin to the optic tectum of various stages of *E. coqui *and *D. pictus *allowed to determine that the first tectofugal fibers develop at stage GH 22 in *D. pictus *and at stage TS 5 in *E. coqui *(data not shown), prior to the ingrowth of retinofugal fibers into the tectum.

### Development of fiber tracts in the brain

**Figure 7:**
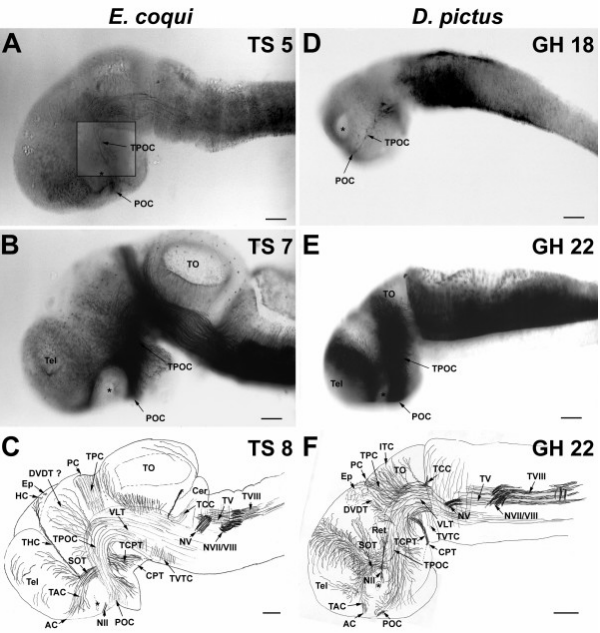
**Development of early fiber tracts in the brain of *E. coqui *(**A**-**C**) and *D. pictus *(**D-F**) as revealed by immunohistochemistry for acetylated tubulin**. Asterisks indicate the optic stalk in all panels. **A**, **D**: The tract of the postoptic commissure and its associated commissure are the first fiber tracts to form in the embryonic forebrain of both species (boxed area in **A **is shown at a more lateral level of focus). **B**, **E**: At midembryonic stages, a more complex scaffold of tracts has formed. **C**, **F**: Camera lucida drawings of embryos with all major fiber tracts indicated. In *E. coqui*, fibers run ventrad along the lateral surface of the thalamus, but no dorsoventral diencephalic tract of tightly bundled fibers is observed (indicated as "DVDT ?"). Abbreviations: AC: anterior commissure; Cer: cerebellum; CPT: commissure of the posterior tuberculum; DVDT: dorsoventral diencephalic tract; Ep: Epiphysis; HC: Habenular commissure; ITC: intertectal commissure; NII: optic nerve; NV: trigeminal nerve; NVII/VIII: facial and vestibulocochlear nerves; PC: posterior commissure; POC: postoptic commissure; Ret: retina (hatched circle); SOT: supraoptic tract; TAC: tract of the anterior commissure; THC: tract of the habenular commissure; Tel: telencephalon; TO: optic tectum; TCC: tract of the cerebellar commissure; TCPT: tract of the commissure of the posterior tuberculum; TPC: tract of the posterior commissure; TPOC: tract of the postoptic commissure; TV: descending tract of the trigeminal nerve; TVTC: tract of the ventral tegmental commissure; TVIII: descending tract of the vestibulocochlear nerve; VLT: ventral longitudinal tract. Bar: 100 μm in all panels.

To relate the timing of retinotectal development to the timing of other differentiation events in the brain, the formation of fiber tracts was studied at various developmental stages of *E. coqui*, *D. pictus*, and *P. pustulosus *in wholemount brains immunostained for acetylated tubulin. Embryonic brain tracts develop similar to zebrafish [38,39] and *Xenopus *embryos [40-42] and were identified using the nomenclature first established in zebrafish embryos. The schedule of development of brain tracts and commissures is given in Table 2 and selected stages are depicted in Fig. 7.

In *E. coqui *and *D. pictus*, three phases of fiber tract development can be recognized (commissures often form slightly later than the associated fiber tracts). In *P. pustulosus*, fiber tract development follows a similar time course, but the earliest phase of brain tract development could not be observed, because no embryos earlier than stage GH 20 were investigated. In the first phase of development (TS 4–5 in *E. coqui*; GH 18 in *D. pictus*; ≤GH 20 in *P. pustulosus*), the tract of the postoptic commissure (TPOC) develops in the forebrain and continues caudad as the ventral longitudinal tract (VLT) (Fig. 7A, B). In the hindbrain, the descending tracts of the trigeminal and vestibulocochlear nerves develop. In the second phase of development (TS 6–7 in *E. coqui*; GH 20–22 in *D. pictus *and *P. pustulosus*), several additional fiber tracts develop in the forebrain and midbrain (tract of anterior commissure, supraoptic tract, tract of posterior commissure, tract of commissure of posterior tuberculum, tract of ventral tegmental commissure, tract of cerebellar commissure) and join the pioneering fibers of the TPOC and VLT. In addition, a discrete dorsoventral diencephalic tract (DVDT) emanating from the epiphysis as a well defined bundle of fibers forms in *D. pictus *and *P. pustulosus *at these stages. In *E. coqu*i, no such distinct bundle of fibers was observed, but fibers running ventrad throughout the thalamus appeared at stage TS 6, a subset of which may correspond to the DVDT. In the third phase of development (≥ TS 8 in *E. coqui*; ≥GH 23–24 in *D. pictus *and *P. pustulosus*), the tract of the habenular commissure joins the axonal scaffold and the intertectal commissure develops. However, at stage TS 9 in *E. coqui *and GH 22 in *D. pictus *only single fibers crossing between the two tectal hemispheres could be observed, indicating that the bulk of the intertectal commissure develops later.

## Discussion

### Derived mode of retinotectal development in *E. coqui*

Direct developing frogs are known from 12 different anuran families [43]. Although phylogenetic relationships among anurans continue to be debated [44-47], the distribution of direct development on any of the proposed anuran phylogenetic trees indicates that it has evolved many times independently from an ancestral biphasic pattern of development.

The development of the retina and tectum in *D. pictus *and *P. pustulosus *as documented here has many similarities with retinotectal development in other biphasically developing frogs including *Rana *[48-51]), *Limnodynastes *[52,53], and *Xenopus*, which is particularly well studied [40,54-68]. Since this pattern of retinotectal development is shared between neobatrachian frogs (*Rana*, *Limnodynastes*, *Physalaemus*), including the leptodactylid frog *Physalaemus *and more basal "archaeobatrachian" lineages such as discoglossids (*Discoglossus*) and pipids (*Xenopus*), it probably represents the primitive condition for extant anurans. Thus, the differences in retinotectal development observed in *Eleutherodactylus *are derived within this direct developing clade of leptodactylid frogs.

In the various biphasically developing species, the different retinal layers develop during embryonic development, when the retina is still small and has reached only a fraction (about one third) of its cross-sectional area at the completion of metamorphosis. The first retinotectal connections are established during early embryonic development, shortly after the major axon tracts have developed in the brain (Tables 1, 2). The expression of *NeuroD *(only described for *Xenopus*), which plays a role in regulating neuronal differentiation and the formation of particular neuronal subtypes such as photoreceptors and amacrine cells in the retina [33,34,37,69-75] becomes restricted to the ciliary margin, the outer part of the inner nuclear layer, and the outer nuclear layer as soon as the different retinal layers develop. Proliferation becomes largely restricted to the ciliary margin at the end of embryonic development, from which the retina then grows during larval stages by addition of cells to all cellular layers.

When the first retinofugal fibers reach the contralateral tectum at late embryonic stages, the latter is still very small and consists predominantly of proliferating cells. Retinal fibers cover the tectum in a rostrocaudal direction during larval stages, as the optic tectum continues to grow by proliferation from the ventricular layer, which is most pronounced caudomedially. At early larval stages, the first tectal layers (layer 7–9 and layer 5) differentiate and retinal fibers cover approximately half of the lateral part of the tectum leaving its midline free of fibers. At this stage the tectum has reached only a fraction (about one third) of its size at the completion of metamorphosis. At the end of metamorphic climax, the entire surface of the tectum is covered by retinofugal fibers. In addition to the main, contralateral projection to thalamus, pretectum, and tectum, an ipsilateral projection to thalamus and pretectum, which subserves important functions for binocular vision in postmetamorphic frogs, begins to develop at late larval stages in *Xenopus *[66,76]. In *D. pictus*, a sparse ipsilateral projection develops already at early larval stages.

Retinotectal development in *E. coqui *has been modified in a number of respects from this ancestral anuran pattern. While the spatiotemporal order of differentiation of retinal and tectal layers and the formation of the retinotectal projection in *E. coqui *have been conserved, patterns of growth and proliferation of retina and tectum have been modified. Both retina and optic tectum grow rapidly during initial formation of the retinotectal projection in embryonic stages so that at a stage when formation of retinal layers and tectal layers 5–9 are completed, they have already reached around 90 % of their size at hatching. While retinal growth in biphasically developing frogs is mostly due to addition of cells from the proliferative ciliary margin, the retina of *E. coqui *grows by proliferation of cells throughout the outer part of the inner nuclear layer of the retina until it has almost reached its size at hatching [29]. Thus, although *NeuroD *expression in the ciliary margin, the outer part of the inner nuclear layer, and the outer nuclear layer resembles the condition in *Xenopus*, many *NeuroD *expressing cells in the inner nuclear layer of *E. coqui *retinae are probably proliferating progenitor cells in contrast to *Xenopus*. *NeuroD *expression in some retinal ganglion cells and absence of *NeuroD *expression in the tectum of *E. coqui *also differ from *X. laevis*, suggesting that some of the functions of *NeuroD *have changed during evolution of *E. coqui*. A sparse ipsilateral retinothalamic projection develops already at embryonic stage TS 9, prior to completion of retinal layer formation and, thus, significantly earlier than in biphasically developing frogs. This may represent an adaptive heterochronic shift related to precocious adoption of postmetamorphic head shape and eye position in *E. coqui*, allowing onset of binocular vision already at hatching stages when the optic tectum is still in a relatively immature, larval-like state (note dissociation between suites XI/XII indicating cranial remodeling and suite II indicating retinotectal differentiation in Fig. 1).

### Dissociation of growth and differentiation during *E. coqui *retinotectal development

The modified pattern of retinotectal development in *E. coqui *indicates that the regulation of growth and differentiation have been dissociated during evolution of *Eleutherodactylus *as discussed in [21] (compare suites III/IV with suite II in Fig. 1). Growth has been greatly accelerated by increased cell proliferation, while the timing of early retinotectal differentiation events has been conserved and remains temporally coordinated with early cranial and spinal development (compare suites IX, V, and II in Fig. 1).

In biphasically developing anurans such as *Xenopus*, thyroid hormones have been implicated in promoting increased proliferation in the retina during mid-larval stages after the thyroid gland develops and thyroid hormone levels rise [77,78]. However, the precocious growth of retina and tectum in *E. coqui *is unlikely to be due to the precocious action of thyroid hormones, because acceleration of retinal and tectal growth is observed from early embryonic stages on, long before the thyroid gland matures and the thyroid axis becomes functional around stage TS 10 in *E. coqui *[30,31]. Although the mechanisms underlying the increase in proliferation in *E. coqui *remain at present obscure, the conserved schedule of retinotectal differentiation implies that increased proliferation is not simply due to a general delay in neural differentiation. Changes in the probability of cell cycle exit or the length of cell cycles are better compatible with the pattern observed.

As a consequence of the rapid growth of retina and tectum during the development of the retinotectal projection, retinofugal fibers in *E. coqui *encounter a much larger target territory as they enter and distribute over the tectum than in biphasically developing frogs. This raises the question, whether the formation of a topographic map of retinal fibers on the tectal surface, as known from other vertebrates, may be compromised in *E. coqui*. However, the formation of retinotopic maps, which is now known to involve gradients of Eph receptors and their ephrin ligands in retina and tectum, is very plastic and can expand or contract in response to drastic experimental reductions of retinal or tectal size, respectively [79,80]. In many fishes and amphibians plastic mechanisms of map formation are important to maintain an ordered retinotopic map throughout development, because their tectum grows in a rostrocaudal direction, while new retinal neurons are added in a radial direction [57,65,81]. This plasticity probably permitted the drastic acceleration of retinotectal growth in *Eleutherodactylus *without compromising the ability to form a well ordered retinotopic map.

Increased proliferation and accelerated growth in *E. coqui *is not confined to the retina and tectum, but is also evident in other parts of the CNS such as the spinal cord [28] and the brain stem (Schlosser, unpublished observation) (compare suites III, IV, and VIII in Fig. 1). This suggests that altered growth patterns in *E. coqui *may be due to systemic regulatory changes affecting proliferation of neural progenitors throughout the CNS, although independent regulatory changes in different parts of the CNS cannot be ruled out.

### Mosaic evolution of CNS development in *E. coqui*

Despite altered growth patterns, the schedules of retinal and tectal differentiation and of the formation of retinotectal projections are conserved and remain coordinated with each other and with the formation of early axon tracts in the brain in *E. coqui *(compare suites II and I in Fig. 1) probaby reflecting their interdependent development for example due to coordinated axon outgrowth. For example, fibers in the tract of the postoptic commissure (TPOC), which is the earliest tract to develop in the embryonic brain, later fasciculate with fibers from multiple other brain tracts as well as with retinofugal fibers. This has led to suggestions that fibers of the TPOC may serve as pioneer axons, on which other brain tracts and retinofugal fibers depend for proper axonal pathfinding [38-41,67,82,83]. However, other studies have shown that retinofugal fibers can properly navigate towards the tectum independent of the TPOC [84,85] suggesting that both TPOC and other fiber tracts including retinofugal fibers may instead follow common pathway cues and, thus, may form in a temporally coordinated fashion once their pathways become established.

Although schedules of retinotectal differentiation and the formation of early brain tracts remain tightly coordinated with each other in *E. coqui*, they have become dissociated from differentiation in other parts of the central and peripheral nervous system. For example, the lateral motor columns (LMCs) in the spinal cord develop precociously in *E. coqui *paralleling precocious development of the limbs, which they innervate [22-25,27,28]. As a consequence, in *E. coqui *LMCs develop simultaneous with the retinotectal system, while in biphasically developing frogs they develop in larval stages after differentiation of most retinal and tectal layers is completed (compare suites VII and II in Fig. 1). Similarly, adult-like cranial skeletal structures and muscles together with their motor innervation precociously differentiate in *E. coqui *in stages [9,13,16,18,19,21], when the retinotectal system is still in an immature, larva-like condition (compare suites XI/XII and II in Fig. 1).

Taken together, this indicates that during evolution of *Eleutherodactylus*, the schedule of differentiation in CNS areas remained coordinated with the schedule of differentiation of those regions in the CNS or in the periphery, with which they are directly connected (retina-tectum-brain tracts, LMC-limbs, cranial motor neurons-cranial muscles), whereas profound temporal dissociations have taken place between structures that are not or only indirectly connected.

## Conclusion

Previous studies have shown that limbs, spinal cord, and the cranial musculoskeletal system develop precociously in direct developing frogs of the genus *Eleutherodactylus*. The present study indicates that retinotectal development was also modified during evolution of *Eleutherodactlyus*. Cell proliferation and growth in retina and tectum have been greatly accelerated in *Eleutherodactylus*, while differentiation of retina, tectum, and fiber tracts in the embryonic brain, which remain temporally coordinated with each other, proceed along a much slower schedule. Consequently, *Eleutherodactylus *hatchlings appear like postmetamorphic froglets with respect to limb, spinal cord, and cranial development and have a correspondingly large brain, while their retinotectal system is still immature and tadpole-like. This suggests that differentiation events in directly interconnected parts of the CNS such as retina and tectum remain coordinated during evolution, while they may be dissociated from proliferation control and from differentiation events in other parts of the CNS probably reflecting the modular character of CNS development.

## Methods

### Animals

Different developmental stages of *Eleutherodactylus coqui *and *Discoglossus pictus *were obtained by natural matings in breeding colonies maintained at the institute. *E. coqui *embryos were staged after Townsend and Stewart (1985) (TS). *D. pictus *embryos were staged after Gallien and Houillon (GH) [86] for embryonic stages and Gosner (Go) [87] for larval and metamorphic stages. Different developmental stages of *Physalaemus pustulosus *were collected at various localities in Costa Rica and staged after [86] for embryonic stages and [87] for more advanced stages. Embryos of *Xenopus laevis *were obtained by in vitro fertilization using standard methods and staged according to Nieuwkoop and Faber (NF) [88]. Larvae and metamorphic stages were anesthesized in tricaine methane-sulfonate (MS 222; Sigma) prior to fixation.

### Immunohistochemistry

For wholemount immunostaining, embryos of *D. pictus*, *P. pustulosus*, and *E. coqui *were fixed in Dent's fixative (80% methanol, 20% dimethyl sulfoxide) and brains were dissected out. Brain tracts were visualized by an anti-acetylated tubulin antibody (6–11B-1; Sigma), which labels all neurites, followed by a peroxidase-coupled secondary antibody and incubation in diaminobenzidine (DAB) as previously described [89,90]. For PCNA immunostaining on sections, embryos and larvae of *D. pictus*, *P. pustulosus*, and *E. coqui *were fixed in Bouin's fixative, dehydrated, paraffin embedded, and serially sectioned (10–15 μm). PCNA immunostaining was performed as previously described [28].

### In situ hybridization

After overnight fixation in 4% paraformaldehyde, embryos and larvae of *E. coqui *and *X. laevis *were dehydrated, paraffin embedded and serially sectioned (10 μm). In situ hybridization with digoxigenin-labeled probes against *E. coqui NeuroD *(*EcNeuroD*, [20]) and *X. laevis NeuroD *(*XNeuroD*, [91]) were performed on paraffin sections following standard procedures [20,92]. The hybridization step was carried out overnight at 55°C and the color reaction was allowed to proceed for 15–30 h. Some sections were counterstained with 1% neutral red.

### Morphometry

The volume of the optic tectum was calculated from measurements of its cross-sectional area in regularly spaced sections through the optic tectum. Cross-sectional area was determined with a graphic table in camera lucida drawings of PCNA-labeled transverse paraffin sections (see above). The entire cross-sectional area (excluding the ventricle), the area of the entire cellular layers (additionally excluding tectal layers 7–9, which are dominated by fibers) as well as the area of the PCNA positive part of the cellular layer were measured in 1–3 individuals per stage. A proliferative index was calculated by dividing the PCNA positive volume by the volume of the entire cellular layer.

### Tracing of retinofugal and tectofugal projections

Tracing studies were performed at different developmental stages of *D. pictus *and *E. coqui*. For tracing of retinofugal projections, the eye was unilaterally extirpated and a biocytin (Sigma) crystal was then applied to the site of lesion. For tracing of tectofugal projections, a biocytin crystal was inserted rostrally into one half of the optic tectum. Larvae and metamorphic stages were anesthesized in tricaine methane-sulfonate (MS 222; Sigma) prior to surgery. After a survival time of approximately 5 hours, animals were fixed in 2% glutaraldehyde/2% paraformaldehyde. After embedding in 4% agar, 50 μm vibratome sections were cut and the tracer was revealed as previously described [19]. Sections were counterstained with nuclear fast red, dehydrated and coverslipped.

## Competing interests

The authors declare that they have no competing interests.
